# Retraction Note: Cerebrolysin Attenuates Exacerbation of Neuropathic Pain, Blood-Spinal Cord Barrier Breakdown and Cord Pathology Following Chronic Intoxication of Engineered Ag, Cu or Al (50–60 nm) Nanoparticles

**DOI:** 10.1007/s11064-026-04785-9

**Published:** 2026-05-16

**Authors:** Hari Shanker Sharma, Lianyuan Feng, Lin Chen, Hongyun Huang, Z. Ryan Tian, Ala Nozari, Dafin F. Muresanu, José Vicente Lafuente, Rudy J. Castellani, Lars Wiklund, Aruna Sharma

**Affiliations:** 1 International Experimental Central Nervous System Injury & Repair (IECNSIR), Dept. of Surgical Sciences, Anesthesiology & Intensive Care Medicine, Uppsala University Hospital, Uppsala University, Frödingsgatan 12, LGH 1103, 75185 Uppsala, Sweden; 2 Department of Neurology, Bethune International Peace Hospital, Zhongshan Road (West), Shijiazhuang, Hebei China; 3 Department of Neurosurgery, Dongzhimen Hospital, Beijing University of Traditional Chinese Medicine, Beijing, 100700 China; 4 Beijing Hongtianji Neuroscience Academy, Beijing, 100143 China; 5 Chemistry & Biochemistry, University of Arkansas, Fayetteville, AR USA; 6 Anesthesiology & Intensive Care, Massachusetts General Hospital, 55 Fruit Street, Boston, MA 02114 USA; 7 Dept. Clinical Neurosciences, University of Medicine & Pharmacy, Cluj-Napoca- Napoca, Romania; 8 “RoNeuro” Institute for Neurological Research and Diagnostic, 37 Mircea Eliade Street, 400364 Cluj-Napoca-Napoca, Romania; 9 LaNCE, Dept. Neuroscience, University of the Basque Country (UPV/EHU), Leioa, Bizkaia Spain; 10 Department of Pathology, University of Maryland, Baltimore, MD 21201 USA; 11 International Experimental Central Nervous System Injury & Repair (IECNSIR), Dept. of Surgical Sciences, Anesthesiology & Intensive Care Medicine, Uppsala University Hospital, Uppsala University, 75185 Uppsala, Sweden

**Retraction Note to: Neurochemical Research (2023) 48:1864–1888**

10.1007/s11064-023-03861-8

The Editor-in-Chief has retracted this article. An investigation by the Swedish National Board for the Assessment of Research Misconduct has determined that one or more elements of data, in the form of tissue images or reported statistical results, was fabricated or falsified [1]. The Editor-in-Chief therefore no longer has confidence in the integrity of the research presented in this article.

The authors did not reply to correspondence from the Publisher about this retraction.
